# Loss to follow-up and associated maternal factors among HIV-exposed infants at the Mbarara Regional Referral Hospital, Uganda: a retrospective study

**DOI:** 10.1186/s12879-020-04964-1

**Published:** 2020-03-19

**Authors:** Rogers Ankunda, Samuel Nambile Cumber, Catherine Atuhaire, Taseera Kabanda, Claude Ngwayu Nkfusai, Frankline Sevidzem Wirsiy, Eleanor Turyakira

**Affiliations:** 1 Faculty of Medicine, Department of Community Health, Mbarara University of Science and Technology, Mbarara, Uganda; 2 Postdoctoral Fellow, Centre for Health Systems Research & Development, University of the Free State, Bloemfontein, South Africa; 3 Office of the Dean, Faculty of Health Sciences, University of the Free State, Bloemfontein, South Africa; 4 School of Health Systems and Public Health, Faculty of Health Sciences, University of Pretoria, Pretoria, South Africa; 5 Faculty of Medicine, Department of Nursing, Mbarara University of Science and Technology, Mbarara, Uganda; 6 Faculty of Medicine, Department of Microbiology, Mbarara University of Science and Technology, Mbarara, Uganda; 7 Cameroon Baptist Convention Health Services (CBCHS), Yaoundé, Cameroon; 8 Department of Microbiology and Parasitology, Faculty of Science, University of Buea, Buea, Cameroon; 9 Faculty of Health Sciences, Department of Public Health and Hygiene, University of Buea, Buea, Cameroon

**Keywords:** HIV-exposed infants, Loss to follow up, Post-natal mother to child transmission

## Abstract

**Background:**

Loss to follow-up (LTFU) deprives HIV-exposed infants the lifesaving care required and results in exposing HIV free infants to virus requisition risk. We aimed to determine the rate of LTFU, postnatal mother-to-child HIV-transmission (MTCT) and to identify maternal factors associated with LTFU among HIV-exposed infants enrolled at Mbarara Regional Referral Hospital PMTCT clinic.

**Methods:**

Study participants were infants born to HIV-positive mothers enrolled in the PMTCT clinic for HIV care at Mbarara Regional Referral Hospital. While access database in the Early Infant Diagnosis (EID) clinic provided data on infants, the open medical record system database at the ISS clinic provided that for mothers. Infants were classified as LTFU if they had not completed their follow-up schedule by 18 months of age. At 18 months, an infant is expected to receive a rapid diagnostic test before being discharged from the PMTCT clinic. Postnatal MTCT of HIV was calculated as a proportion of infants followed and tested from birth to 18 months of age. Logistic regression was used to determine possible associations between mothers’ characteristics and LTFU. In-depth interviews of mothers of LTFU infants and health workers who attend to the HIV-exposed infants were carried out to identify factors not captured in the electronic database.

**Results:**

Out of 1624 infants enrolled at the clinic, 533 (33%) were dropped for lack of mother’s clinic identification number, 18 (1.1%) were either dead or transferred out. Out of 1073 infants analysed, 515 (48%) were LTFU by 18 months of age while out of the 558 who completed their follow-up schedule, 20 (3.6%) tested positive for HIV. Young age of mother, far distance to hospital and non-use of family planning were identified as outstanding factors responsible for LTFU. In addition, in-depth interviews revealed facility-level factors such as “waiting time” which would not be found in routine client databases.

**Conclusion:**

This study has revealed a high rate of loss to follow up among HIV-exposed infants enrolled at Mbarara Regional Referral hospital PMTCT clinic. Young maternal age, long distance to health facility and failure to use family planning were significantly associated with LTFU. Incorporating family planning services in the ART and PMTCT clinics could reduce loss to follow-up of HIV exposed infants. Young mothers should be targeted with information on the importance of completing the EID follow-up schedule and also, their clinic identification number be gotten at each visit.

## Background

The impact of HIV on children cannot be over emphasised. The 2013 global statistics revealed that 3.2 million children under 15 years old, 9.1% of the global population, were living with the virus [1]. In sub- Saharan Africa lives 91% of children with the virus with vertical transmission a leading cause of infant infection [2], 6% of HIV infected children are in Asia and the Pacific and the remaining 3% live in other parts of the world [1]. By a 2013 appraisal, 1.3 million women living with HIV were delivered of their babies without any change from 2009 [1]. A significant drop of mother-to-child transmission rate of HIV from 25.8% in 2009 to 16% (13–18%) in 2013 was recorded [1]. Annually, nearly 1.4 million HIV-1 positive women conceive, majority of whom are from sub-Saharan Africa [2]. The proportion of women living with HIV-1 among antenatal clients in sub-Saharan Africa ranges from 5% to as high as 30%.

In Uganda, there are 1.2 million HIV infected people with 57% made of females and 13% children while new infections were estimated at 124,000 [3]. In 2012, the prevalence of HIV among adults was at 7.2% with 780,000 women and 190,000 children living with HIV respectively. In Uganda, 160,000 children became infected with HIV and 328 HIV related deaths were reported every day [4]. The 2010 PMTCT Annual Report projected that in 2010, 1,550,000 pregnancies occurred increased to 1,600,000 in 2011 in Uganda. This could be translated into 100,750 and 104,000 pregnant women living with HIV in 2010 and 2011 respectively [3].

HIV-1 can be transmitted from mother-to-child during pregnancy, labor and delivery and after birth through breastfeeding [5]. The risks of viral transmission during pregnancy ranges from 5 to 10%, during labor and delivery from 10 to 20%, and during mixed infant feeding from 10 to 20%. In the absence of any intervention to prevent mother- to-child transmission (MTCT), it is estimated that the viral transmission risk will rise rapidly from 15 to 45%. Therefore, with effective interventions, this risk can be reduced to levels below 5% [6]. Over the years, there have been milestone research and developments in knowledge and interventions that can mitigate and save lives of infants exposed to HIV Virus.

The World Health Organization highly recommend for HIV-exposed infants to be tested using HIV-1 DNA PCR test. Yet, in 2013, only 42% of infants born to mothers living with HIV in low and middle-income countries received this test within 2 months as recommended. In Uganda, only 36% of HIV-exposed infants were tested [1] mainly because they were not brought for follow-up care in Early Infant HIV Diagnosis (EID) clinics.

The failure of HIV exposed infants to return for follow-up care puts those born free of HIV (whose 1st PCR was negative) at risk of acquiring HIV and deprives those who have already acquired the virus the necessary care they need to stay alive.

In more than a week period, over 48% of infants receiving EID services at Mbarara Regional Referral Hospital miss their appointments. This study sought to determine the proportion of loss to follow-up and identify maternal factors associated with loss to follow-up among HIV exposed infants enrolled at Mbarara Hospital PMTCT clinic (unpublished EID registers, 2012).

## Methods

### Study design

This was a mixed study design comprising a descriptive retrospective registry-based cohort study and In-depth Interviews. The retrospective study was carried out on infants born to HIV positive mothers attending the PMTCT/EID clinic at Mbarara Hospital Immune Suppression Syndrome (ISS) clinic. In addition, qualitative interviews of mothers whose babies were lost to follow-up and health workers in the EID/PMTCT clinic were carried out.

### Study setting

The study was conducted at the ISS clinic of Mbarara Regional Referral Hospital (MRRH) in Mbarara district. Mbarara district is found in the south-western part of Uganda, and is located 270kms from Kampala city. At this hospital like in all maternal child health (MCH) facilities, all pregnant women attending antenatal care (ANC) clinic receiving counselling, are tested for HIV and are recorded in PMTCT or ANC registers. All HIV positive women are recorded in PMTCT care registers and instantly placed on lifelong ART treatment regardless of CD4 count or gestation period. They are started on ART combination of TDF, 3TC and EFV. Infants born to mothers infected by HIV are documented in the EID register after birth, and followed up till they are 18 months old and within this time, mothers receive counselling on infant feeding along with ARV prophylaxis for PMTCT of HIV. To this effect, infants whose mothers are placed on lifelong ART are given once daily NVP from birth to 6 weeks of age regardless of whether they are exclusively breastfed or given replacement feeding and thereafter receive cotrimoxazole prophylaxis. According to the Uganda HIV infant testing algorithm, an HIV DNA PCR test is carried out at ≤6 weeks of age and cotrimoxazole is then started. In the case where the 1st DNA PCR is negative and the child has been breastfeeding, the 2nd DNA PCR test is carried out 6 weeks after breastfeeding has stopped. When a 2nd PCR turns out to be negative, a rapid HIV test is conducted at 18 months old prior exit of care by child. On the other hand, if the 1st or 2nd PCR is positive, the infant is referred for ART initiation. By June, 2014, the EID/PMTCT clinic at Mbarara Hospital ISS clinic had 3120 infant’s cumulative enrolment since its inception in 2005, and by January 2013, 2072 infants enrolled.

### Study population

The study was delimited to HIV exposed infants who enrolled for care at the Mbarara Hospital PMTCT/EID clinic between January 2010 and January 2013 and whose mothers received care from Mbarara Regional Referral Hospital ISS clinic during the same period. Infants whose mothers’ lacked clinic identification numbers were deemed ineligible.

### Sample size estimation

All HIV-exposed infants who had been enrolled from January 2010 to January 2013 and followed up for 18 months and whose mothers received care from ISS clinic were analysed. A sample size of 772 mother-infant pairs was estimated based on assumptions of 80% power; odds ratio for loss to follow up of 2.2 [7] and level of significance of 0.05 (two-sided). This estimate was reached at using a method in observational epidemiology for calculating sample size for unmatched cross-sectional studies, cohort studies and randomized clinical trials [7, 8].

### Quantitative data collection

Data on HIV-exposed infants were obtained from the Access database system for the PMTCT/EID clinic whereas, data on mothers were obtained from the electronic Open Medical Record System (Open MRS), the database for the ISS clinic. Data of eligible mother-infant pairs were identified by unique client numbers in the electronic databases and the dataset converted into STATA format (Stata Corporation Inc.). The main outcome variable was loss to follow-up among HIV-exposed infants. An infant was classified loss-to follow-up if he/she did not complete follow up to the point of being discharged and was not declared deceased. Data on independent variables were extracted as recorded in databases and later categorised where necessary as described by Kabakyenga and colleagues (1). For example, age was categorised and coded as *0 “18–23”, 1 “24–29” and 2 “> 30 years”.*

### Qualitative data collection

Fourteen interviews were conducted, ten of them with selected HIV positive mothers whose exposed infants were lost to follow up and four with health workers who attend to these infants. The purpose of the qualitative interviews was to capture factors associated with LTFU that could have been missed out in the electronic database of the ISS clinic such as facility-level client experiences and health worker behaviours. Tracing of mothers of lost babies was done by phone calls after their information had been generated from the data set. Only mothers who had left their phone contacts with the clinic were contacted. Interviews were audio recorded, transcribed word verbatim and translated approximately from the local dialect to English language for analysis.

### Data analysis

Quantitative data analysis was done using Stata version 11.0 (Stata Corp, College Station, TX, USA). Frequency counts and percentages were obtained to describe socio-demographics and other categorical variables. The median and inter-quartile ranges were used to describe quantitative variables with skewed distributions such as CD4 count. In the bivariate analysis, independent variables were cross tabulated with the outcome variable to determine possible associations. Odds Ratios and their 95% confidence intervals were calculated. Independent variables with *p-value* ≤ *0.2* in the bivariate analysis were entered into a multivariable logistic regression model to adjust for confounding. Statistical significance of variables in the final model was assessed based on a *p*-value threshold of ≤*0.05*. In analysing the qualitative data, English transcripts were read in between the lines to identify codes and themes. Identification of codes was guided by the conceptual framework and study objectives with room for emergent themes from the data. Coding and analysis were done manually using a cut and paste approach where segments from the transcripts were copied and assigned to the generated codes.

## Results

### Characteristics of study participants

Between January 2010 and January 2013, there were 1624 infants registered in the EID clinic at Mbarara Regional Referral hospital. One third of these infants (33%) were excluded from analysis because the mother’s ISS clinic identification number to link the infant and mother’s data was missing in the EID record. Also, 1.6% of the remaining 1091 infants, was left out because they were dead or moved to other clinics i.e., they did not contribute towards obtaining the desired outcome. A cohort of 1073 mother-infant pairs enrolled into PMTCT and followed up for 18 months was therefore analysed **(**Table 1**).**

**Table 1:** Characteristics of Study Participants

Characteristics	Total Cohort (*n* = 1073)	LTFU (*n* = 515)	Discharged (*n* = 558)
Age (median IQR)	26 (23–30)	26 (22–30)	27 (24–30)
Religion: n (%)			
Catholic	266 (24.8)	133 (25.8)	133 (23.8)
Protestant	465 (43.3)	224 (43.5)	241 (43.2)
Muslim	107 (9.9)	47 (9.1)	60 (10.7)
Other	40 (3.7)	24 (4.6)	16 (3.1)
Missing	195 (18.2)	87 (16.9)	108 (19.4)
Marital Status: n (%)			
Single	81 (7.5)	39 (7.5)	42 (7.5)
Married	706 (65.8)	348 (67.6)	358 (64.2)
Separated	115 (10.7)	52 (10.1)	63 (11.3)
Divorced	53 (4.9)	24 (4.7)	29 (5.2)
Widowed	71 (6.6)	32 (6.2)	39 (6.9)
Missing	47 (4.4)	21 (4.1)	26 (4.7)
Education: n (%)			
None	30 (2.8)	16 (3.1)	14 (2.5)
Primary	533 (49.7)	269 (52.2)	264 (47.3)
Secondary	224 (20.9)	111 (21.5)	113 (20.3)
Tertiary	62 (5.8)	23 (4.5)	39 (6.9)
Missing	224 (20.9)	97 (18.9)	127 (22.8)
Parity (Median IQR)	2 (1–4)	2 (1–4)	2 (1–4)
Income: n (%)			
< 100,000	622 (57.9)	302 (58.6)	320 (57.3)
100,001-250,000	101 (9.4)	46 (8.9)	55 (9.8)
250,001-500,000	18 (1.7)	9 (1.7)	9 (1.6)
> 500,000	10 (0.9)	4 (0.8)	6 (1.1)
Missing	322 (30.0)	154 (29.9)	168 (30.1)
Distance: n (%)			
Short	529 (49.3)	243 (47.2)	286 (51.3)
Long	315 (29.4)	165 (32.0)	150 (26.9)
Missing	229 (21.3)	108 (20.9)	121 (21.7)
Occupation: n (%)			
Unemployed	227 (21.2)	120 (23.3)	107 (19.2)
Student	18 (1.7)	8 (1.5)	10 (1.8)
Civil Servant	29 (2.7)	13 (2.5)	16 (2.9)
Business	209 (19.5)	93 (18.1)	116 (20.8)
Armed forces	2 (0.2)	1 (0.2)	1 (0.2)
Farmer	316 (29.4)	160 (31.1)	156 (27.9)
Other	152 (14.2)	67 (13.0)	85 (15.2)
Missing	120 (11.2)	53 (10.3)	67 (12.0)
Alcohol Consumption: n (%)			
No	706 (65.8)	338 (65.6)	368 (65.9)
Yes	170 (15.8)	84 (16.3)	86 (15.4)
Missing	197 (18.4)	93 (18.1)	104 (18.6)
Disclosure: n (%)			
No	70 (6.5)	33 (6.4)	37 (6.6)
Yes	772 (71.9)	373 (72.4)	399 (71.5)
Missing	231 (21.5)	109 (21.2)	122 (21.9)
Family planning use:			
No	200 (18.6)	112 (21.8)	88 (15.8)
Yes	873 (81.4)	403 (78.2)	470 (84.2)

### Proportion of HIV-exposed infants lost to follow-up

Out of the total cohort of 1073 infants analysed, 515 (48%; 95% CI: 45 to 51%) were LTFU by age 18 months; they did not complete follow-up to the point of being discharged and were not declared deceased. The findings revealed that lost to follow-up was 261 (24%) at 3 months, 308 (29%) at 6 months while 436 (40.6%) and 515 (48%) were lost to follow up at 12 and 18 months into care respectively **(**Table 2**).**

**Table 2:** Proportion of loss to follow up of infants against time

Time of LTFU	Cumulative LTFU n (%)	Active n (%)	95% CI
At 3 months	261 (24.3)	812 (75.7)	0.217–0.269
At 6 months	308 (28.7)	765 (71.3)	0.260–0.314
At 12 months	436 (40.6)	637 (59.4)	0.376–0.436
At 18 months	515 (48.0)	558 (52.0)	0.450–0.510

### Proportion of mother to child transmission (sero-conversions)

Out of the 515 infants who were LTFU, 7 infants (1.4%) had tested positive for HIV and 508 had an unknown HIV status (had negative 1st or 2nd PCR results but didn’t return for final testing). Of the 558 who were discharged, 20 infants (3.6%) tested positive for HIV and were referred for care into the ART clinic.

### Factors associated with loss to follow up of HIV-exposed infants

Mother’s age, distance from home to hospital and family planning use were found to be associated with loss to follow up following the bivariate analysis conducted. Compared to mothers within the ages 18–23 years, *p* = 0.002 (OR = 0.6; 95% CI 0.44–0.82), mothers with age > 30 years were least likely to have their infants lost to follow up. Again, those with 25–29 years were less likely to have their infants lost to follow up compared to mothers with 18–23 years of age, *p* = 0.05 (OR = 0.57; CI 0.42–0.76). Long distances from home that took mothers more than 1 h to get to the hospital were associated with infant LTFU, *p = 0.05* (OR = 1.31, 95% CI 0.99–1.73). Infants whose mothers were not using any family planning method were more likely to be lost to follow up, *p = 0.01* (OR = 1.48, 95% CI 1.09–2.02).

Multivariable analysis indicated that young age of mother; long distance from home to hospital; and failure to use family planning methods were independently associated with loss to follow-up **(**Table 3**).** Compared to those infants whose mothers were in the 18–23 years age category, the odds of LTFU were lower among those whose mothers fell in the 24–29 age category. The odds of loss to follow-up were higher among babies whose mothers were reported to come from faraway places to hospital at enrolment in ISS clinic compared to those whose mothers were coming from near.

**Table 3:** Bivariate and Multivariate analysis of factors associated with loss to follow up of HIV exposed infants

Characteristics	Total Cohort (*n* = 1073) LTFU: n (%)	Crude OR(95% CI)	*P*-Value
Bivariate analysis			
Age			
18–23	322 (30.0)	1[Reference]	
24–29	438 (40.8)	0.57 (0.42–0.76)	0.002*
> 30	312 (29.1)	0.60 (0.44–0.82)	
Religion			
Christian	771 (71.8)	1[reference]	
Muslim	107 (9.9)	0.80 (0.53–1.20)	0.28
Marital Status			
Single	320 (29.8)	1[Reference]	
Married	706 (65.8)	1.14 (0.87–1.48)	0.34
Education			
None	30 (2.8)	1[Reference]	
Primary	533 (49.7)	0.88 (0.42–1.85)	0.75
Secondary/Tertiary	286 (26.6)	0.77 (0.36–1.64)	0.50
Parity			
Prima-parity	101 (9.4)	1[Reference]	
Multi-parity	222 (20.7)	0.85 (0.53–1.36)	0.49
Income			
< 100,000	622 (57.9)	1[Reference]	
100,001-250,000	101 (9.4)	0.87 (0.58–1.35)	0.58
> 250,000	13 (1.2)	0.92 (0.43–1.96)	0.84
Distance			
Near (< 1 h away)	535 (49.8)	1[Reference]	
Far (> 1 h away)	322 (30.0)	1.31 (0.99–1.73)	0.05*
Disclosure			
No	70 (6.5)	1[Reference]	
Yes	772 (71.9)	1.05 (0.64–1.17)	0.85
Alcohol Consumption			
No	706 (65.8)	1[Reference]	
Yes	170 (15.8)	1.06 (0.76–1.49)	0.72
Family planning use			
Yes	873 (81.4)	1[Reference]	
No	200 (18.6)	1.48 (1.09–2.02)	0.01*
Multivariate analysis			
Age			
18–23	322 (30.0)		
24–29	438 (40.8)	0.53 (0.38–0.74)	
> 30	312 (29.1)	0.56 (0.39–0.81)	
Distance			
Near (< 1 h away)	535 (49.8)		
Far (> 1 h away)	322 (30.0)	1.49 (1.11–2.00)	
Family planning use			
Yes	873 (81.4)		
No	200 (18.6)	1.83 (1.29–2.60)	

*Statistically significant, *CI* Confidence Interval

The odds of loss to follow-up were higher among babies whose mothers reported they did not use family planning compared to those whose mothers were using family planning at enrolment in ISS clinic **(**Table 3**).**

### Findings from in-depth interviews

Factors identified from qualitative interviews were grouped facility-level and client barriers.

### Facility level factors

#### Waiting time

Mothers pointed out waiting time as one of the reasons why they did not return their babies to the facility for follow-up. One respondent had this to say:

*“The other thing is that we spend a lot of time here. You try to come early so that by midday you are going back home but you find yourself returning home in the evening. When it comes to lunch time, the health workers close and go for lunch, by the time they come back, time has already elapsed and yet you wanted to go look for some money to feed the children*,” The young mother explained.

Waiting time was also mentioned by one health worker as a hindrance to child follow-up. A nurse who works with the HIV-exposed infants’ clinic when quizzed on why mothers were not returning their children for follow-up, submitted that: *“It might be the long queue at the clinic especially for working class mothers.”*

### Client based factors

#### Distance to health facility

Travelling long distances to the health facility was reported as a barrier to adhering to EID clinic schedules. A mother from Bushenyi had this to say:

*“I come all the way from Bushenyi with a child. We spend a whole day here with nothing for my child to eat. Sometimes, the health workers are slow and by the time we are out of the queue, the child is already very hungry. So, when I thought about the long journey to and from the health facility and how very challenging it is, I decided to leave my child at home since I was not breastfeeding and the child’s first results were negative,”* she justified.

### Short childbirth spacing and multiple visits from single household

When children are born too close to each other, this can affect their HIV care because the burden of carrying two children to care is enormous for the mother. This is worsened if mother and children have different follow-up dates.

*“Like I said, my second child came a bit too early after the first. So, the first didn’t come to the clinic the number of times she was supposed to have come. She came in once. But the second one because she was sick, I had to bring her, and then bring myself, and then bring her … you couldn’t ask for the same day, you would get disturbed … being called here and there. So that was challenging. When my daughter was still sick, I had to bring her every month, then bring myself as well. But when she got better, that kind of changed. That’s why I didn’t even bother bringing her for discharge,”* the young mother explained.

## Discussion

The outcome of this research reveals that loss to follow-up rates (24% at 3 months, 29% at 6 months, 40.6% at 12 months and 48% at 18 months) among infants who were enrolled for PMTCT services between January 2010 and January 2013 were progressively high. This finding could be attributed to the existing socio-demographic, socio-economic and behavioural characteristics of mothers. In addition, facility-based factors could also have influenced this high LTFU rate. Compared to that reported in a similar cohort in Western Kenya by [9] which reported a rate of 27.4% by 18 months follow–up, the rate of LTFU in this study cohort is higher. The relatively low MTCT rate in infants who complete follow-up (3.6%) could be justified by the new option B+ treatment guidelines for prevention of mother-to-child transmission of HIV introduced by the Ministry of Health of Uganda. According to this option, pregnant women who test HIV-positive are immediately placed on life time ART on their first antenatal visit. The constant radio adverts on PMTCT may also have contributed to this reduced transmission rate considering that in the [3] country report, vertical transmission of HIV was reported to account for 20% of all HIV infections in Uganda. Comparative to rates reported in Tanzania and Ethiopia, the rate of MTCT in this study cohort is low. Mwendo and colleagues [10] reported a 9.6% MTCT rate in Kilimanjaro region, Tanzania. The variation in rate of transmission between this study and the one carried out in Tanzania could be attributed to accessibility of health services by study participants in both studies. Majority of the mothers in this study were urban dwellers whereas participants in the study in Tanzania were mainly rural residents who were more likely to face difficulty in accessing PMTCT services. While Berhan and colleagues [11] reported 10.1% HIV prevalence among HIV exposed infants in a facility-based study carried out in Amhara region, Ethiopia, the variation in rate of transmission between these two studies could be attributed to country-level differences in response to elimination of MTCT. Whereas Uganda has a HIV prevalence rate among pregnant women of 6.1% [12] compared to 1.2% of Ethiopia, the MTCT rate is lower in Uganda at 20% [3] compared to 33% in Ethiopia [11].

These researchers found out that among mother-infant pairs receiving HIV care at Mbarara Regional Referral hospital PMTCT clinic, young maternal age, travelling long distances to the hospital and failure to use family planning were independently associated with loss to follow-up. In Uganda, the median age at first marriage among women within the ages 25–49 years is 18.2 years. Early marriage and early pregnancy coupled with HIV infection brings a lot of stress to young women who would still be girls and in school under normal circumstances. Although early marriage and pregnancy is a common occurrence in Uganda, there is still a lot of stigma and young women of school going age find it hard to seek family planning services. Young age of the mother has been found to be associated with LTFU in previous studies in Mali and Malawi [13, 14]. Whereas parity was not significantly associated with loss-to-follow-up, qualitative results indicated that number of children a woman has to take for EID services may be an important factor influencing loss-to-follow-up. Failure to use family planning leads to short childbirth spacing and multiple visits from a single household especially if scheduling of clinic visits is not family-centred. Mothers may also find it difficult to carry more than one child to the hospital for every appointment thereby leading to LTFU. Again, walking long distances to access a health facility was significantly associated with LTFU. This was confirmed in the qualitative interviews where mothers revealed that lack of transport and long distances to health facility were barriers to completing follow-up. This may be attributable to unavailability of PMTCT services at lower health centres. Stigma may also be one of the contributing factors to why women travel long distances and seeking PMTCT services.

Qualitative data revealed a complex interplay between facility-level and client-level barriers. For example, the influence of long distance to the health facility on infant follow-up may be compounded by delays at the health facility due to long waiting time and the mother’s inability to afford out-of-pocket expenses for lunch during clinic visits. In addition, qualitative data suggests that mothers’ decisions to take or not to take their infants for EID follow-up visits may be influenced by their own interpretation of initial negative PCR results amidst the search for reasonable manoeuvres to minimise client burden of seeking HIV care.

### Limitations

The researchers used routine data from the ISS clinic database and some information was missing. Thirty three percent of the eligible study participants were dropped due to lack of mothers’ clinic identification numbers which made it impossible to find their data in the ISS client database. This in part reflects the poor data recording practices in hospital settings which could affect validity of results from analyses of routine patient care data. If missingness of mother’s client ID was in some way related to the LTFU, it means that our results could be an under-estimate of LTFU rate. However, we do not have a reason to assert that this was the case. While it may be reasonable to be cautious with interpretations of our quantitative results, we note that they are well corroborated by qualitative data.

The quantitative analyses utilised variables routinely collected during patient care and did not consider other important factors such as waiting time, behaviour of health workers and information held by mothers regarding care for exposed infants. However, through in-depth interviews, the researchers were able to highlight some of the important variables not found in electronic databases.

Due to lack of enough funds to carry out tracking of lost infants, these researchers only interviewed mothers who responded to phone calls and managed to travel to the clinic for the interviews. The researchers could have missed out on a number of factors affecting mothers in their homes. A thorough investigation of causes of LTFU and sero-conversion rates of HIV exposed infants is warranted to provide stronger evidence for design and funding of effective programs to reduce LTFU.

Despite these limitations however, we firmly assert that the study findings are useful to inform implementation of PMTCT in large ART clinics in Uganda and other comparable settings.

## Conclusion

This research paper reveals a high rate of loss to follow up among HIV-exposed infants enrolled at Mbarara Hospital PMTCT clinic. Young maternal age, long distance to health facility and failure to use family planning were meaningfully associated with LTFU. Short childbirth spacing, child wellbeing, lack of transport, lack of partner support, client waiting time, and limited waiting space at health facility, behaviour of health workers, and bridge of confidentiality may also influence LTFU. In a bid to eliminate mother-to-child HIV transmission and enhance subsequent care and treatment for HIV exposed infants, strategies for retaining children who are at the greatest risk of morbidity and mortality in care need to address issues identified in this study.

Our findings suggest incorporating family planning services in the ART and PMTCT clinics could reduce loss to follow-up of HIV exposed infants. Young mothers should be targeted with information about the importance of keeping their babies’ clinic appointments even when their babies are not ill in order to reduce children’s risk of infection and also provide the necessary care to those already infected.

### Recommendation

Since 33 % (33%) of the eligible study participants were dropped due to lack of mothers’ clinic identification numbers which made it impossible to find their data in the ISS client database, we therefore recommend that, at every visit of the mother to the clinic, the health care provider to ensure that identification numbers be given to all pregnant women who come to the hospital for clinic.

## Data Availability

De-identified data that support the findings of this study are available from the first author, Rogers Ankunda but restrictions apply under license for the current study. The data may be made publicly available upon reasonable request and with permission of ISS clinic located in Mbarara Regional Referral Hospital, Mbarara, Uganda.

## Abbreviations

ANC: Antenatal clinic
EID: Early Infant Diagnosis
HIV: Human Immunodeficiency Virus
LTFU: Loss to follow-up
MRRH: Mbarara Regional Referral Hospital
MTCT: Mother to child HIV-transmission
PCR: Polymerase Chain Reaction
PMTCT: Prevention of Mother-to- Child HIV-transmission
